# Exploring the Resistance Mechanisms of Distal D835V Mutation in FLT3 to Inhibitors

**DOI:** 10.1155/2022/3720026

**Published:** 2022-03-28

**Authors:** Zhiwei Wang, Baichun Hu, Yu An, Jian Wang

**Affiliations:** ^1^Department of Pharmaceutical Chemistry, Jinzhou Medical University, No. 40 Section 3 Songpo Road, Linghe District, Jinzhou 121001, China; ^2^Key Laboratory of Structure-Based Drug Design and Discovery, Ministry of Education, Shenyang Pharmaceutical University, 103 Wenhua Road, Shenhe District, Shenyang 110016, China; ^3^Department of Open Education, Jinzhou Open University, No. 9 Section 7 Jiefang Road, Linghe District, Jinzhou 121001, China

## Abstract

**Objective:**

FMS-like tyrosine kinase 3 (FLT3) is an attractive therapeutic target in acute myeloid leukemia. Unfortunately, secondary FLT3 mutations that developed resistance to inhibitors have become a severe problem. Specifically, ASP-835 (D835F/H/V/Y) mutant within the activation loop of FLT3 is the most commonly encountered drug-resistant and observed secondary FLT3 mutations. In this study, we carried out a set of computational approaches to explore how this mutation influenced the conformation and dynamics of DFG motif in a manner altered inhibitors' susceptibility.

**Methods:**

Molecular dynamics (MD) simulation, dynamic cross-correlation (DCC) analysis, surface area (SASA), binding free energy (MM-GBSA), and structural analysis were used to compare the severe and minor D835V mutation-induced impact to sorafenib and crenolanib, respectively.

**Results:**

The A-loop of the FLT3 protein may experience conformational change in the presence of the resistant mutation, which were mainly positioned at PHE-830. The protein-inhibitor interactions displayed that the motions of PHE-830 influenced that of sorafenib, but not to crenolanib.

**Conclusions:**

These findings indicated that the structural impact brought by D835V mutation should be considered in designing novel drugs to overcome resistance to FLT3-D835V.

## 1. Introduction

Acute myeloid leukemia (AML) is a malignant hematological tumor derived from ongoing cell proliferation of hematopoietic progenitor cells. These cells lose their ability to give rise to functional blood cells, followed by the accumulation of myeloblasts within the bone marrow [1]. Mutations in the FMS-like tyrosine kinase 3 (FLT3) gene occur in approximately 30% of the patients with AML and may play the role of driver mutations of this disease. Therefore, FLT3-activating mutations become an attractive therapeutic target in AML [2].

FMS-like tyrosine kinase 3 (FLT3), belonging to a subfamily of class III receptor tyrosine kinases (RTKs), is primarily expressed by hematopoietic cells and plays an irreplaceable role in the stem cell development and immune system [1]. It consists of an extracellular domain, a transmembrane domain, a juxtamembrane domain (JM), and a highly conserved kinase domain (KD) interrupted by a kinase insert domain. Griffith et al. first reported the crystal structure of the autoinhibited form of FLT3, which was composed of the juxtamembrane domain and kinase domain [3]. The global architecture of kinase domain resembles most protein kinases, with a smaller N-terminal domain (N-lobe) and a larger C-terminal domain (C-lobe). The N-lobe contains five-stranded *β* sheet and an *α* helix, named helix *α*C, while seven *α* helices and three *β* strands form C-lobe. The N- and C-lobes are linked by a flexible polypeptide stretch (a hinge region), which permit proper rotational movement within the two domains relative to each other.

FLT3 functioned as a molecular switch could adopt two extreme conformations: “on” state and “off” state, which correspond to active and inactive conformation, respectively. The shift from an “on” to “off” form involves crankshaft-like motions of ASP-PHE-GLY (DFG) motif in the N-lobe of the activation loop [3, 4]. In the “on” state, the N-terminal lobe undergoes a rotation toward the C-terminal lobe which result in a DFG-in orientation of DFG motif, and the activation loop adopts the open form that is beneficial for the binding of ATP and protein substrates. Conversely, if the N-terminal lobe undergoes a rotation away from the C-terminal lobe and folds into a cleft to close the open conformation, thereby the DFG motif is a DFG-out orientation that is not compatible with ATP binding site and protein substrates, which is associated with an inactive conformation of the kinase.

**Figure 1:**
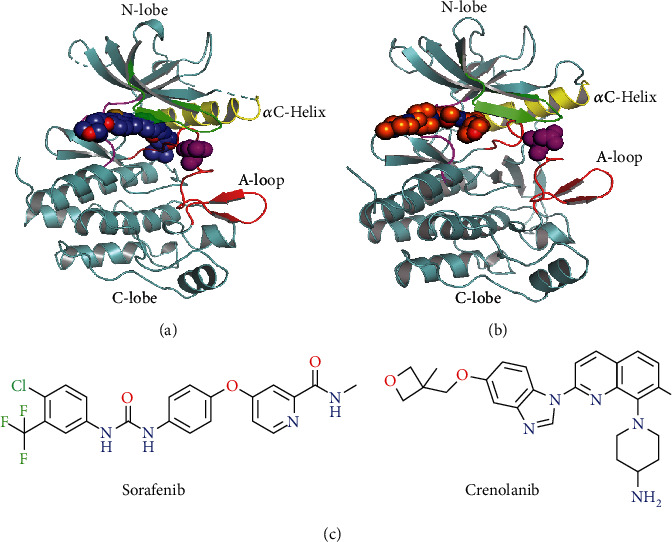
The structural features to differentiate the active and inactive states of FLT3. (a) Crystal structure of inactive FLT3 (PDB code: 4RT7); the cocrystallized ligand is shown in slate sphere. (b) Crystal structure of active FLT3 (PDB code: 6JQR); the cocrystallized ligand is shown in orange sphere. Both in (a) and (b), the D835V mutations are shown in magenta sphere. (c) 2D chemical structures of sorafenib and crenolanib.

A number of potent small-molecule inhibitors targeting FLT3 kinase have been investigated as potential therapeutic for the treatment of AML. These inhibitors include quizartinib (AC220, approved for AML by PMDA), sorafenib (DB00398), midostaurin (PKC412, approved for AML by FDA), gilteritinib (ASP2215, approved for AML by FDA), sunitinib (SU11248), lestaurtinib (CEP-701), crenolanib (CP-868596), and PLX3397 (pexidartinib) [5–13]. Unfortunately, secondary FLT3 mutations observed in adults and children, containing N676K/D, N841 (I/T/Y), A627T, G697R, F691L, D835/H/Y/V, Y693C, D698N, and N701K, that developed resistance to inhibitors have become a severe problem [14–19]. Specifically, amino acid exchanges at D835 (D835F/H/V/Y) within the activation loop of FLT3 are the most commonly encountered drug-resistant and observed secondary FLT3 mutations. These substitutions are unfavorable for the stability of the FLT3-inactive conformation required for the binding by type II inhibitors [17]; however, the FLT3-active conformation bound to type I inhibitors is free from the influence of these variants. This study contrasted type II inhibitor sorafenib with type I inhibitor crenolanib to state the fact of the drug resistance mechanisms of FLT3-D835V (Figure 1).

Sorafenib as a biaryl urea compound targets all the members of Raf protein kinases, PDGFR, VEGF 2/3, and c-Kit kinases [20]. It is also a type II inhibitors with activity against FLT3 [17, 21]; however, on account of the inhibitor capacity is weaker than the kinase activity, it displays limited activity against secondary FLT3-D835 mutations. Crenolanib (CP-868596) is a benzamidine quinolone derivative that was initially developed as a platelet-derived growth factor receptor (PDGFR) inhibitor but also displays high affinity with other class III RTKs, for example, FLT3 [22, 23]. Two reports suggests the characterization of it as a novel type I TKI that harbors activity against FLT3 including ITD and/or D835-activating mutants in vitro [11, 12] and in vivo [12].

In this work, molecular dynamics (MD) simulations were performed to investigate the different mechanisms of the two promising inhibitors (sorafenib and crenolanib), which were resulted from D835V mutation. Then, dynamical cross-correlation (DCC) analysis, binding free energy, and structural analysis were applied to reveal the effect of the D835V mutation on the flexibility and inhibitors binding. We believed these illuminating results would enhance our comprehension of D835V resistance mechanisms, and the structural information may be useful for the future development of novel inhibitors to overcome the D835V mutation of FLT3.

## 2. Materials and Methods

### 2.1. Protein and Ligand Preparation

To date, crystal structures for only two FLT3-WT are available from the Protein Data Bank, and those are inactive and active conformation of FLT3 (PDB code: 4RT7 and 6JQR), respectively [24, 25]. By comparison of FLT3 amino acid sequences as well as crystal structures analysis, we identified a missing loop (831-836) in 6JQR. Although other active conformations of FLT3 have not yet been reported, the crystal structure of KIT (PDB code: 1PKG), determined in an active conformation, shares 56% identity with FLT3 KD (Figure S1) [26]. Hence, we added the missing loop for active FLT3 using the KIT structure as the template by the SWISS-MODEL online server (https://www.swissmodel.expasy.org) [18]. Before docking, the FLT3 proteins were prepared using the ProteinPrep Wizard from Schrodinger software package (version 2014), including removing the crystallographic water molecules, building missing loops, and adding hydrogen atoms. After that the ionization states of all amino acid residues were settled at pH 7.4. Finally, the OPLS_2005 force field unit was performed to optimize the protein complexes. The structures of sorafenib and crenolanib were sketched in ChemAxon Marvin Sketch and converted to three-dimensional conformation by LigPrep wizard in the Schrodinger software package (version 2014).

### 2.2. Docking Studies

To build the initial complex of 4RT7-WT+sorafenib, 4RT7-D835V+sorafenib, 6JQR-WT+crenolanib, and 6JQR-D835V+crenolanib, we utilized the protein-ligand docking module (Glide) in Schrödinger. To search for the possible regions of ligands in the binding site of the receptor, Glide carried out a series of filters. Firstly, a grid with several different sets of fields was generated and supplied more accurate scores for the ligands poses; then through the extensive conformational search, conformational flexibility of ligands was handled in Glide, which rapidly eliminated unsuitable conformations; finally, Schrödinger's proprietary GlideScore scoring function was applied to rescore the energy-minimized poses. To identify ligand poses with unfavorable energies, the extra precision (XP) mode of Glide performed a custom scoring function and combined a powerful sampling protocol, which were used to screen out active compounds with available poses and favorable scores for appropriate hydrophobic interactions, H-bonding, and other contacts [27]. Noting that, appropriate protein and ligand preparations played particularly critical role in the XP Glide scoring function. The all-atom structures with appropriate bond orders and formal charges, named as “prepared” structures, were used to generate the receptor grid, which was performed in the receptor grid generation panel. The prepared protein-ligand complex was defined as the receptor, and the cocrystallized ligand was used to define the active site, whose size was 10 Å × 10 Å × 10 Å (inner box). Ligand docking jobs would be applied after the generation of receptor grids. The extra precision (XP) with the default parameters was selected as the docking precision using prepared receptor grid and ligands. The top scoring docked models (4RT7-WT+sorafenib, 4RT7-D835V+sorafenib, 6JQR-WT+crenolanib, and 6JQR-D835V+crenolanib), well separated from others in terms of the docking scores (Table S1), were selected to represent the most favorable complex and discussed in the Results and Discussion sections. As a reference, the second scoring docked models were also maintained and displayed in Supplementary Materials. All of these complex structures were used for the subsequent molecular dynamics (MD) simulations to investigate the dynamic features. Pymol (version 1.7.2.1) was used to produce the pictures elucidating the protein-ligand interactions.

### 2.3. Molecular Dynamics (MD) Simulation

The top and second scoring docked models (4RT7-WT+sorafenib, 4RT7-D835V+sorafenib, 6JQR-WT+crenolanib, and 6JQR-D835V+crenolanib) were subjected to one hundred nanoseconds molecular dynamics simulations on Desmond (v3.8) module within Schrödinger suite [28]. A simple point charge (SPC) water model was embedded in the initial structures, which were neutralized by moderate number of counter ions to keep the concentration of physiological salts at about 0.15 M, and the system was set in an orthogonal box. After that, the minimization jobs were carried out with OPLS_2005 force field to relax the system. Then, the whole system was minimized using the hybrid method of the steepest decent and the limited-memory Broyden-Fletcher-Goldfarb-Shanno algorithms (LBFGS) with the maximum of 5000 steps until the value of gradient threshold was reached to 25 kcal/mol/Å. Finally, 100 ns MD simulations were performed, applying a normal pressure temperature (NPT) [29, 30]. Moreover, analyzing root mean square deviation (RMSD), root mean square fluctuation (RMSF), and protein-ligand interactions during the whole simulation could obtain the degree of stability of protein-inhibitor complex and key amino acid residues participating in the contact with the inhibitors.

### 2.4. Dynamic Cross-Correlation Map (DCCM)

In order to calculate the dynamic residue movement, GROMACS was used to generate the dynamic cross-correlation map (DCCM) on the C*α* atoms of the protein using the MD trajectory [31, 32]. The cross-covariance matrix was used to describe the correlated motions of residues, and the range of that was from -1.0 to 1.0, which means completely anticorrelated motions and completely correlated motions, respectively.

### 2.5. Binding Free Energy Calculation

In order to forecast the binding free energies of inhibitors with FLT3, the MM-GBSA method was used through Prime module of Schrödinger suite that was also an important method in assessing the docking result. The mean conformations extracted from the 100 ns MD simulations were minimized, and the energies of the FLT3 inhibitors are calculated with OPLS_2005 force field [33]. The calculating formula is as follows: Δ*G*_bind_ = Δ*G*_complex_ − (Δ*G*_protein_ + Δ*G*_ligand_), where Δ*G*_bind_ is the representative of the binding free energy, Δ*G*_complex_ means the free energy of the complex, Δ*G*_protein_ is marked as the free energy of FLT3 protein, and Δ*G*_ligand_ stands for the free energy of the inhibitors.

## 3. Results

### 3.1. MD Stability of the Simulated Systems

**Figure 2:**
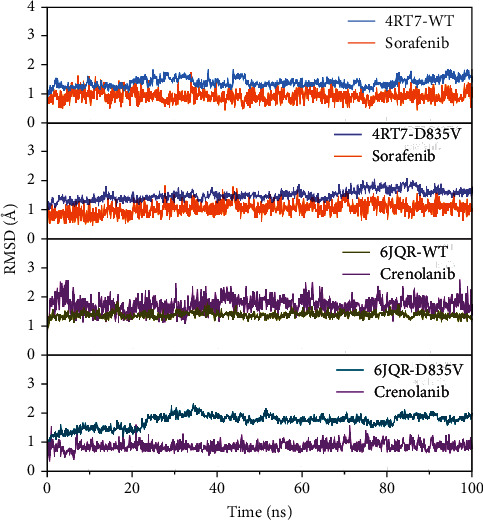
RMSD curves of C*α* atoms in the protein backbone and heavy atoms of ligands during MD simulations.

Root mean square deviation (RMSD) values revealed the dynamic motion of C*α* atoms in the protein backbone and heavy atoms of ligands and provided insight into the stability of the simulated complexes. The plotted RMSD curves (Figure 2) manifested that the RMSD curves in all systems reached at equilibrium after approximately 10-40 ns and maintained their stability until the end of the simulation.

**Figure 3:**
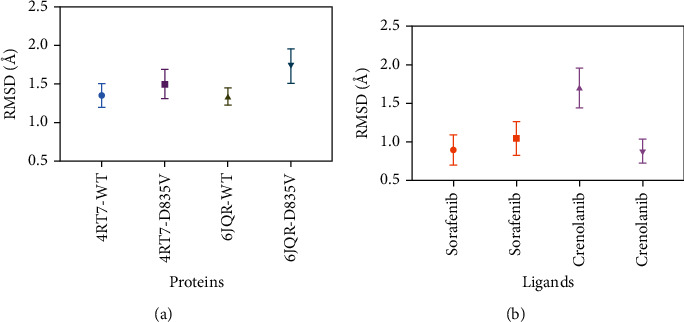
The average RMSD values of C*α* atoms in the protein backbone and heavy atoms of ligands during MD simulations.

As shown in Figure 3, the RMSD values of FLT3-D835V were a bit larger than those of the FLT3-WT for most of the simulation. The RMSD curves for 4RT7-WT and 4RT7-D835V oscillated with minute fluctuations (less than 0.5 Å) and showed medium RMSD with average RMSD values of 1.36 Å and 1.50 Å, respectively. The RMSD curve for 6JQR-D835V C*α* atoms of the protein backbone was the most active one with minute fluctuations (less than 0.5 Å) and exhibited the highest average RMSD value of 1.74 Å. In contrast, the 6JQR-WT C*α* atoms of protein backbone exhibited the most stable RMSD curve, with a lower average RMSD of 1.35 Å. The heavy atoms of sorafenib in 4RT7-WT and 4RT7-D835V oscillated with lower fluctuations in their respective RMSD, with average RMSD values of 0.89 Å and 1.04 Å, respectively, while the RMSD curves for crenolanib in 6JQR-WT and 6JQR-D835V were interesting. The former was steeper with a higher average RMSD, but the latter was flatter with a lower average RMSD (1.69 Å versus 0.87 Å, respectively).

### 3.2. Interregional Correlated Motions of the Simulated Systems

**Figure 4:**
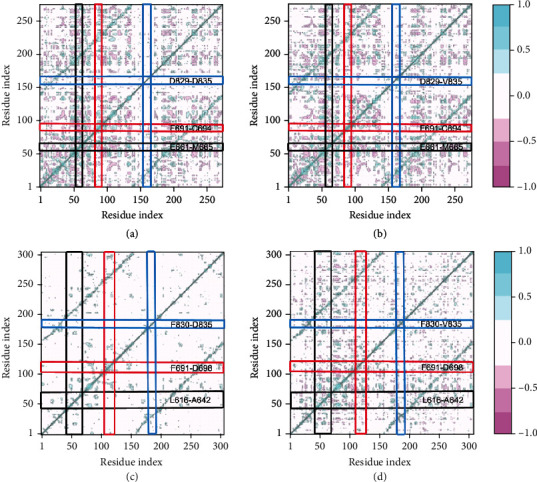
DCCM from MD simulations. (a) 4RT7-WT+sorafenib; (b) 4RT7-D835V+sorafenib; (c) 6JQR-WT+crenolanib; and (d) 6JQR-D835V+crenolanib.

To probe the influence of D835V mutant on the dynamic motions of the FLT3 protein, the residue cross-correlation analysis was calculated for the four systems, whose results were depicted in Figure 4. The blue color regions represented positive correlated motions of residues with values ranging from 0.5 to 1, while the pink color regions was associated with the anticorrelated movements, and the corresponding values were ranged from -0.5 to -1; and the light pink and blue color regions were related to values ranging from -0.5 to +0.5 and showed weak or no correlated motions.

The residues at positions of 57-61 and 87-90 in 4RT7-WT, respectively, generated almost the same anticorrelated motions as that of 4RT7-D835V, while residues at positions of 152-158 in 4RT7-WT produced weaker correlated motions than that of 4RT7-D835V (Figures 4(a) and 4(b)). Residues at positions of 44-70, 119-126, and 188-193 in 6JQR-WT experienced strongly positive correlated motions; comparing with 6JQR-WT, two of them (residues at positions of 44-70 and 188-193) in 6JQR-D835V showed a decreased level of correlated motions, and the remaining one (residues at positions of 119-126 in 6JQR-D835V) formed weak anticorrelated motions (Figures 4(c) and 4(d)).

**Figure 5:**
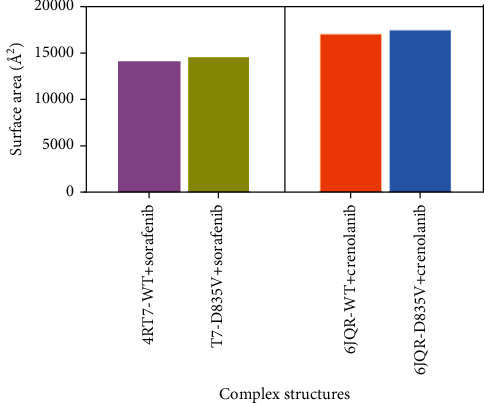
The solvent accessible surface area of four complex structures.

Solvent accessible surface area (SASA) is a key feature of proteins for considering as a decisive factor in their folding and stability. It could be defined as the surface surrounding a protein, characterized by a hypothetical solvent-ball center with a van der Waals contact surface of the molecule. In this study, we calculated solvent SASA to emphasize the accessibility of proteins to solvent. As shown in Figure 5, the SASA of 4RT7-D835V+sorafenib (14675.45 Å^2^) increased 3.76% compared to that of 4RT7-WT+sorafenib (14,143.89 Å^2^), which indicated that 4RT7-D835V bound with sorafenib increased the stability of the protein. As a comparison, the proportion of SASA change for 6JQR-WT and 6JQR-D835V bound with crenolanib (17,108.31 Å^2^ versus 17,541.86 Å^2^, respectively) was smaller (2.53%), which was in accordance with RMSDs and DCCMs. However, these conformational changes were observed in all complexes.

### 3.3. Binding Free Energies Predicted by MM-GBSA

**Figure 6:**
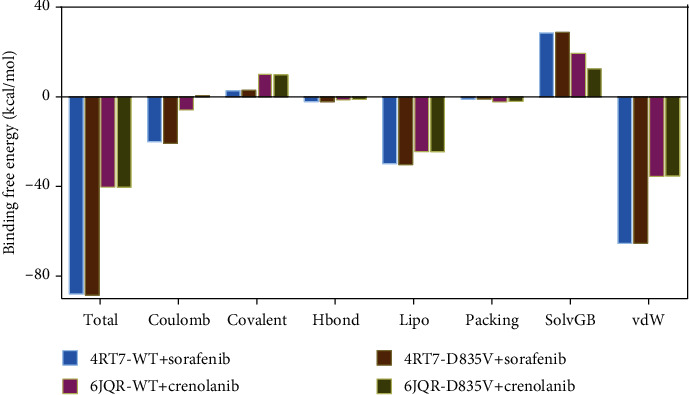
Binding free energies of 4RT7-WT+sorafenib (blue), 4RT7-D835V+sorafenib (brown), 6JQR-WT+crenolanib (violet), and 6JQR-D835V+crenolanib (green). The total energy is composed of several different types of interactions, including coulomb, covalent, H-bond, lipophilic, packing, SolvGB, and vdW interactions.

Subsequently, the binding free energies of 4RT7-WT+sorafenib, 4RT7-D835V+sorafenib, 6JQR-WT+crenolanib, and 6JQR-D835V+crenolanib were calculated by MM-GBSA method and listed in Figure 6 and Supplementary Materials Table S2. As shown in Figure 6, Δ*G*_bind_ were composed of seven types of interactions, containing coulomb, covalent, H-bond, lipophilic, packing, SolvGB, and vdW interactions. The Δ*G*_bind_ of 4RT7-D835V was a little higher than that of 4RT7-WT (-88.562 kcal/mol versus -88.081 kcal/mol, respectively), when they bound with sorafenib, while 6JQR-WT+crenolanib had a larger Δ*G*_bind_ of -40.593 kcal/mol than that of 6JQR-D835V+crenolanib (-40.461 kcal/mol).

### 3.4. The Protein Fluctuations and Structural Changes Caused by the D835V Mutant

**Figure 7:**
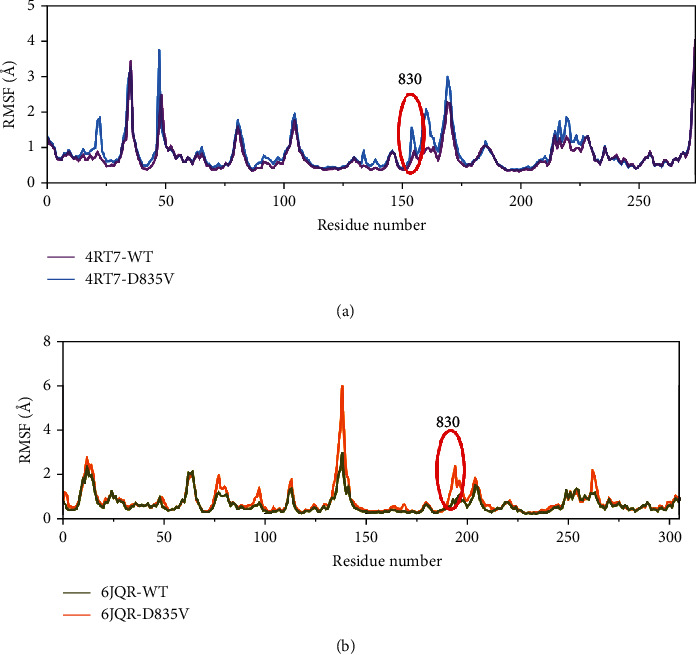
RMSF for the C*α* atoms of FLT3. (a) 4RT7-WT (violet) and 4RT7-D835V (blue) and (b) 6JQR-WT (green) and 6JQR-D835V (orange).

Root mean square fluctuation (RMSF) of the C*α* atoms in all residues was calculated to evaluate the protein residues related to the differences in behavior observed between FLT3-WT and FLT3-D835V. To study the mobile region induced by D835V mutant, the RMSF analyses acquired from 100 ns MD simulation explained the fluctuations of each residue, and low RMSF values proposed that all the residues displayed stability in their corresponding dynamic state during MD simulation. As displayed in Figure 7, the plots of the RMSF values showed that residues in FLT3-D835V protein structure experienced higher fluctuations than those in FLT3-WT protein structure. The observed fluctuations are mainly limited at the positions of 830 and 835 for FLT3-D835V in comparison with FLT3-WT, which fluctuated the most frequently than others.

### 3.5. Elucidate the Binding Modes of Inhibitors with FLT3 by Molecular Dynamics Simulation

**Figure 8:**
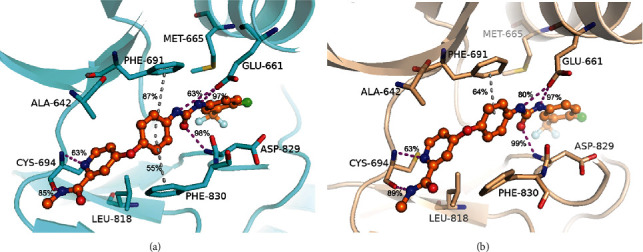
MD-predicted structures of the 4RT7-sorafenib complexes. (a) 4RT7-WT+sorafenib and (b) 4RT7-D835V+sorafenib. 4RT7-WT backbone is shown as cyan cartoons, and 4RT7-D835V backbone is shown as wheat cartoons. Sorafenib is shown as orange ball-sticks. Magenta-dashed lines represent hydrogen bonds, and gray-dashed lines represent Pi-Pi stacking interactions.

**Figure 9:**
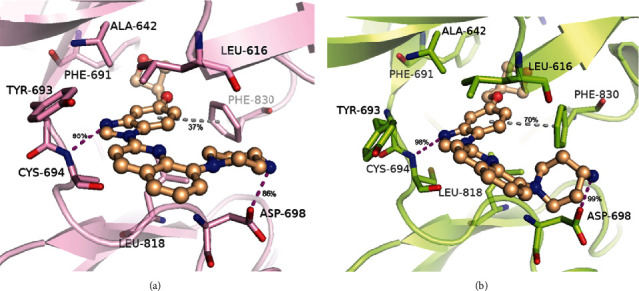
MD-predicted structures of the 6JQR-crenolanib complexes. (a) 6JQR-WT+crenolanib and (b) 6JQR-D835V+crenolanib. 6JQR-WT backbone is shown as violet cartoons, and 6JQR-D835V backbone is shown as lemon cartoons. Crenolanib is shown as wheat ball-sticks. Magenta-dashed lines represent hydrogen bonds, and gray-dashed lines represent Pi-Pi stacking interactions.

In order to better explain the resistance mechanism of the inhibitors to D835V mutant of FLT3, we conducted to analyze the type of major interactions associated with the key residues during the simulations of FLT3-WT and FLT3-D835V proteins (Figures 8 and 9).

As shown in Figure 8(a), the *N*-methylpicolinamide “head” fitted the adenine pocket, interacting two H-bonding contacts with CYS-694. The urea linker between the phenyl moiety and 1-chloro-2-(trifluoromethyl)-benzene interacted favorably with both GLU-661 and ASP-829, which were found in other type II inhibitors including urea linker, such as quizartinib [25]. The phenyl ring, positioned in the middle of the cavity, realized edge-to-face contacts with both PHE-691 (gatekeeper) and PHE-830 (of the DFG motif). Except for these electrostatic interactions, sorafenib stabilized the complex by van der Waal forces through its surrounding hydrophobic residues. The 1-chloro-2-(trifluoromethyl)-benzene “tail,” which was a hydrophobic moiety, filled the bottom of the allosteric back pocket and participated in hydrophobic contacts with MET-665. The pyridine ring interacted with hydrophobic residues ALA-642 and LEU-818. These similar interactions were also found in the CDK8 kinase and p38a kinase, which bound with sorafenib [34, 35]. In Figure S2, we examined the interactions of second scoring docked models of sorafenib which reduced several signature interactions. This comparison supported that the top scoring docked models could represent the most favorable 4RT7-WT+sorafenib complex. As shown in Figure 8(b), the conserved H-bonding contacts and steric interactions of sorafenib were also observed in 4RT7-D835V protein simulations. However, the proportion of edge-to-face contact with gatekeeper PHE-691 decreased from 87% to 64%, and the other edge-to-face contact with PHE-830 was notably absent, which caused substantial loss in binding affinity. These data verified that ASP-835 was a key residue of A-loop, determining the conformational state of the DFG motif and the orientation of PHE-830.

Figure 9(a) highlighted the binding mode of crenolanib in 6JQR-WT, which was characterized by a benzimidazole-quinoline scaffold with 4-amine-1-piperidin fragment attached to 8-position of quinoline and a substituted benzyloxy moiety linked to 5-position of benzimidazole, respectively. The quinoline ring pointed outward from the entrance adenine binding pocket. The benzimidazole ring is inserted in the adenine binding pocket and formed H-bonding contacts with lateral chain of CYS-694. This H-bond occurrences were nearly 95% among the MD simulation. Moreover, the binding interaction was mainly hydrophobic. Specifically, residues LEU-616, ALA-642, PHE-691, and CYS-693 (of the N-terminal lobe) induce van der Waal interactions from the upper face of benzimidazole-quinoline moiety, whereas residues LEU-818 (of the C-terminal lobe) and PHE-830 (of the conserved DFG motif) formed hydrophobic packing contacts with the benzimidazole-quinoline moiety to further stabilize the binding between crenolanib and 6JQR-WT. In this pose, PHE-830 also formed aromatic interactions with benzimidazole moiety in crenolanib, while the occurrences was only 30%. In addition, the 4′-amine attached to the piperazine ring interacted with the main-chain carbonyl group of ASP-698 (86%). However, we did not find any interactions between substituted benzyloxy moiety and the surrounding residues. Our predicted crenolanib binding mode in 6JQR-WT was consistent with the binding of crenolanib to c-Kit kinase [18], while simulations of second docked models (Figure S3) revealed that the disappeared interactions might weaken the binding affinity of crenolanib to 6JQR-WT. The preserved binding of crenolanib in 6JQR-D835V was shown in Figure 9(b). The benzimidazole-quinoline moiety stabilized the complex via van der Waal interactions that involved residues LEU-616, ALA-642, PHE-691, CYS-693, LEU-818, and PHE-830 from the upward and downward. The benzimidazole moiety and 4-amine-1-piperidin fragment each connected with CYS-694 and ASP-698 through H-bonds, with occurrence values of 98% and 99%, respectively. Surprisingly, the aromatic contact between the phenyl of benzimidazole moiety and PHE-830 was also observed, with the occurrence up to 70%.

## 4. Discussion

Previous studies have demonstrated that D835V mutation in FLT3 conferred clinical resistance to sorafenib but had no effect on crenolanib. However, the exact impact of this distal mutation in FLT3 is not wholly understood. In this study, we carried out a set of computational approaches to explore how this mutation influenced the conformation and dynamics of DFG motif in a manner altered inhibitors' susceptibility.

MD simulations showed that there were minute fluctuations in the dynamic motion of the FLT3-D835V with respect to the FLT3-WT, which could be concluded that the overall structural rigidity of FLT3 maybe decreased by the D835V mutant. While the gently RMSD curves for sorafenib in 4RT7-WT and 4RT7-D835V indicated that greater resistance of sorafenib to the MD force field, in contrast, the fluctuated RMSD curves for crenolanib in 6JQR-WT and 6JQR-D835V showed the structural flexibility of crenolanib during the MD simulation. Moreover, lower RMSD values (less than 2 Å) in all systems may be attributed to more stable conformations of the complex.

The results of DCCM analysis were in accordance with those obtained from MD simulations. The 4RT7-WT displayed similar dynamic motions as those in 4RT7-D835V; the positive correlated motions were mostly observed in the diagonal region (blue), and the anticorrelated movements mainly focused on the off-diagonal region (pink). While the resulting DCCMs between 6JQR-WT and 6JQR-D835V clearly displayed the distinct motions, 6JQR-WT experienced strongly positive correlated motions, and 6JQR-D835V showed a decreased level of correlated motions. The analysis of the overall SASA of the different complexes revealed the conformational changes induced by D835V mutant might be the main factor for energy redistribution.

By analyzing the Δ*G*_bind_ between the same inhibitor and FLT3-WT or FLT3-D835V, we did not discover any meaningful differences. It prompted us to investigate the reason in detail. Analysis of the interactions between FLT3 (WT and D835V mutant) and inhibitors, which were showed with the percentage of duration and displayed in a histogram in Figure S4 and S5, revealed that there were almost no differences in the proportion of major interactions formed between FLT3 and key residues.

However, based on the histogram shown in Figure 6, we could find that the Δ*G*_bind_ values rose dramatically from the inactive type to the active type of FLT3. Sorafenib showed higher affinity to 4RT7-WT and 4RT7-D835V, while crenolanib showed lower affinity to 6JQR-WT and 6JQR-D835V. Further in-depth analysis of Δ*G*_bind_ values between the two types of conformation was carried out. Notably, Δ*G*_bind_ of vdW interactions accounted for the largest portion to binding affinity for 4RT7-WT+sorafenib and 6JQR-WT+crenolanib, with the values of -65.600 kcal/mol and -35.460 kcal/mol, respectively. As for 4RT7-D835V+sorafenib and 6JQR-D835V+crenolanib, Δ*G*_bind_ of vdW interactions were also the highest obstruction to binding affinity (-65.550 kcal/mol versus -35.447 kcal/mol, respectively).

**Figure 10:**
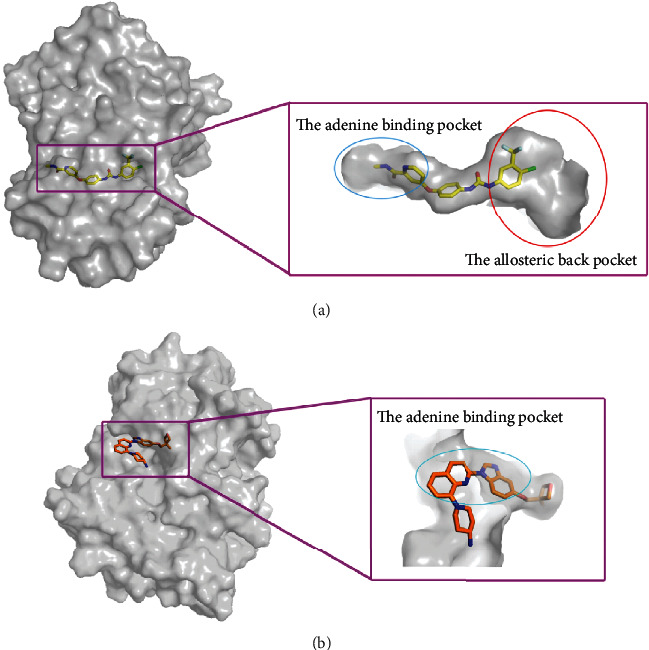
(a) The binding pocket of sorafenib in 4RT7-WT and (b) the binding pocket of crenolanib in 6JQR-WT. The protein is shown in gray. Sorafenib is shown in yellow sticks, and crenolanib is shown in orange sticks.

We explored, by docking studies, the conformations of sorafenib and crenolanib, representative of two kinds of binding modes within the active site. It could be found from Figure 10(a) that sorafenib embedded in the binding pocket. It adopted the canonical type II inhibitor binding mode, fitting perfectly the adenine binding pocket and the allosteric back pocket. As indicated in Figure 10(b), unlike type II inhibitors which wedged between the adenine and allosteric back pockets, crenolanib interacted favorably in the adenine binding pocket. Hence, the entire binding site of crenolanib was relatively outward compared with that of sorafenib, which explained the differences of vdW interactions in Δ*G*_bind_ between the active type and the inactive type of FLT3.

**Figure 11:**
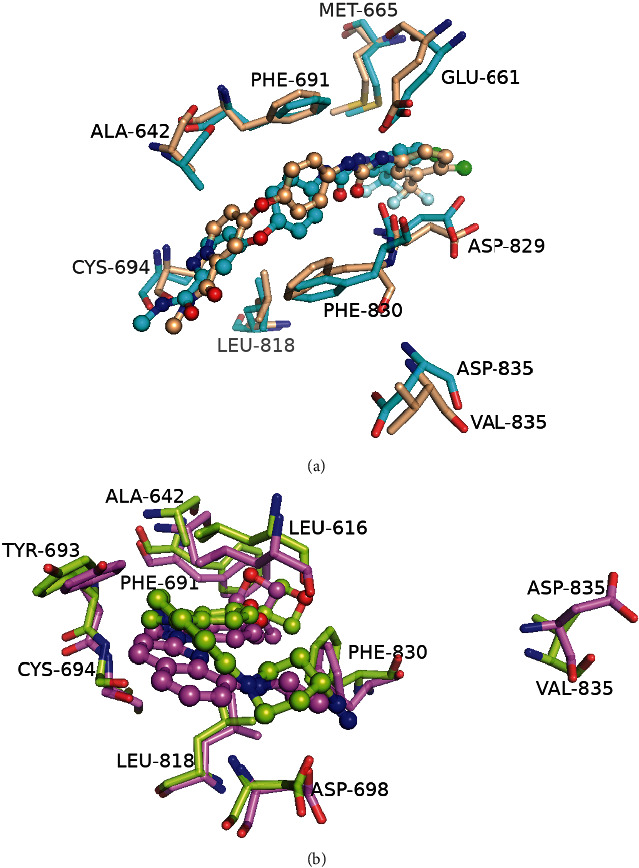
Alignment of the binding pocket between (a) 4RT7-WT+sorafenib (cyan) and 4RT7-D835V+sorafenib (wheat) and (b) 6JQR-WT+crenolanib (violet) and 6JQR-D835V+crenolanib (lemon).

Compared with FLT3-WT, the observed fluctuations in FLT3-D835V obtained by RMSF plot glide to further analyze the tertiary structures of the WT and D835V mutant (Figure 11). Given that residue ASP-835 (D835) of the A-loop was too far away from the adenine binding pocket and the allosteric back pocket to be able to interact directly with inhibitors binding in these pocket. Compared with FLT3-WT, in FLT3-D835V promt us to further analyze the tertiary structures of the WT and D835V mutant (Figure 11). Given that the residue ASP-835 (D835) of the A-loop was too far away from the binding pocket to be able to interact directly with inhibitors. But the findings obtained from RMSF clearly showed that PHE-830 of the conserved DFG motif underwent the conformational change in the D835V mutant protein. Moreover, we could verify that the observed conformational change had led to residue PHE-830 via electrostatic or steric interactions to influence binding affinity for type I or type II inhibitors.

Taken together, the results of RMSD, DCCM, SASA, MM-GBSA, RMSF, and structural analysis were in agreement with those obtained from docking studies. Analyzing the binding modes of sorafenib revealed that the motions of it were associated with that of residue PHE-830 affected by D835V mutation, which explained why D835V mutation in FLT3 conferred clinical resistance to sorafenib. However, the effect of D835V mutant on sorafenib binding could not be seen on that of crenolanib. These data demonstrate that crenolanib retained its activity against sorafenib-resistant FLT3 D835V mutation.

## 5. Conclusions

To our knowledge, no studies have explored the resistance mechanisms of distal D835V mutation in FLT3 to sorafenib and crenolanib. Through a set of computational approaches, we found that D835V mutation may increase the flexibility of the global FLT3-WT related to conformational changes which were mainly limited at PHE-830. These findings may contribute to design novel potent inhibitors in the future, which could avoid the structural impact brought by D835V mutation.

## Data Availability

This work was performed at the Computer-Aided Drug Design Center of Shenyang Pharmaceutical University.

## Supplementary Materials

Supplementary Materials Supplementary materials include raw data of the sequence alignment between 6JQR and 1PKG, glide docking scores of sorafenib and crenolanib, calculated binding free energies for four complexes, MD-predicted structure of the second scoring 4RT7-WT+sorafenib complex and 6JQR-WT+crenolanib complex, 4RT7 (WT and D835V) contact with sorafenib, and 6JQR (WT and D835V) contact with crenolanib.
