# Supplementary material for: Exploring the Resistance Mechanisms of Distal D835V Mutation in FLT3 to Inhibitors
Source: Oxid Med Cell Longev. 2022 Mar 28;2022:3720026. doi: 10.1155/2022/3720026 (PMC8979743; doi:10.1155/2022/3720026)
Supplement: Supplementary Materials — include raw data of the sequence alignment between 6JQR and 1PKG, glide docking scores of sorafenib and crenolanib, calculated binding free energies for four complexes, MD-predicted structure of the second scoring 4RT7-WT+sorafenib complex and 6JQR-WT+crenolanib complex, 4RT7 (WT and D835V) contact with sorafenib, and 6JQR (WT and D835V) contact with crenolanib. [file 3720026.f1.pdf]

Supplementary Materials

Zhiwei Wang,[1] Baichun Hu,[2] Yu An[3] and Jian Wang[2,*]

[1] Department of Pharmaceutical Chemistry, Jinzhou Medical University, No.40 Section 3 Songpo Road, Linghe District, Jinzhou 121001, China;

[2] Key Laboratory of Structure-Based Drug Design and Discovery, Ministry of Education, Shenyang Pharmaceutical University, 103 Wenhua Road, Shenhe District, Shenyang 110016, China;

[3] Department of Open Education, Jinzhou Open University, No.9 Section 7 Jiefang Road, Linghe District, Jinzhou 121001, China;

[*] Corresponding author. Tel./ fax: +86 24 52430227/+86 24 23995043 (J. Wang);

e-mail: jianwang@syphu.edu.cn (J. Wang).

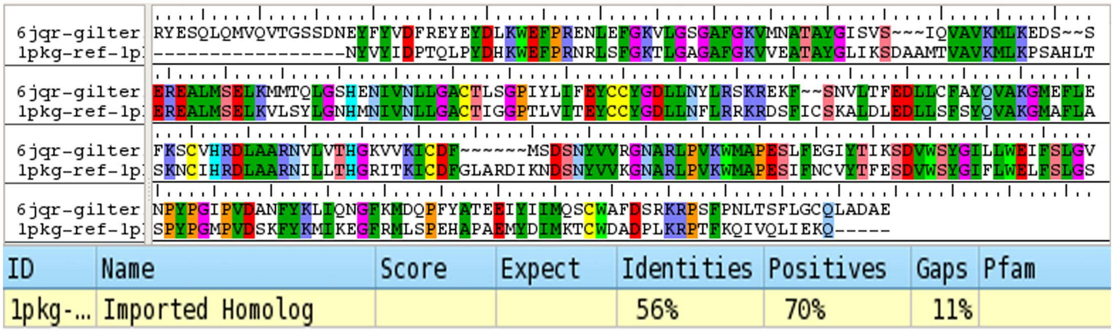

FIGURE S1: The sequence alignment was made between 6JQR and 1PKG.

TABLE S1: Glide docking scores of sorafenib and crenolanib.

Complex	Glide gscore (Kcal/mol)
4RT7-WT+sorafenib-1	-11.284
4RT7-WT+sorafenib-2	-8.234
4RT7-D835V+sorafenib-1	-11.276
4RT7-D835V+sorafenib-2	-7.695
6JQR-WT+crenolanib-1	-8.267
6JQR-WT+crenolanib-2	-4.322
6JQR-D835V+crenolanib-1	-9.575
6JQR-D835V+crenolanib-2	-6.377

TABLE S2: Calculated binding free energies (kcal/mol) of 4RT7-WT+sorafenib, 4RT7-D835V+sorafenib, 6JQR-WT+crenolanib and 6JQR-D835V+crenolanib.

	4RT7-WT+ sorafenib	4RT7-D835V+ sorafenib	6JQR-WT+ crenolanib	6QJR-D835V+ crenolanib
Total	-88.08071804	-88.56204995	-40.59271112	-40.46072342
Coulomb	-20.17925543	-20.93800849	-6.21237005	0.556820886
Covalent	2.799853996	3.020685053	9.95198492	9.967485008
Hbond	-2.403109563	-2.401548628	-1.305829015	-1.308400561
Lipo	-30.02138012	-30.45143371	-24.66384354	-24.64845542
Packing	-1.266053253	-1.141289674	-2.236116557	-2.239760127
SolvGB	28.58926649	28.88586619	19.33332149	12.65888228
vdW	-65.60004016	-65.54957663	-35.45985837	-35.44729549

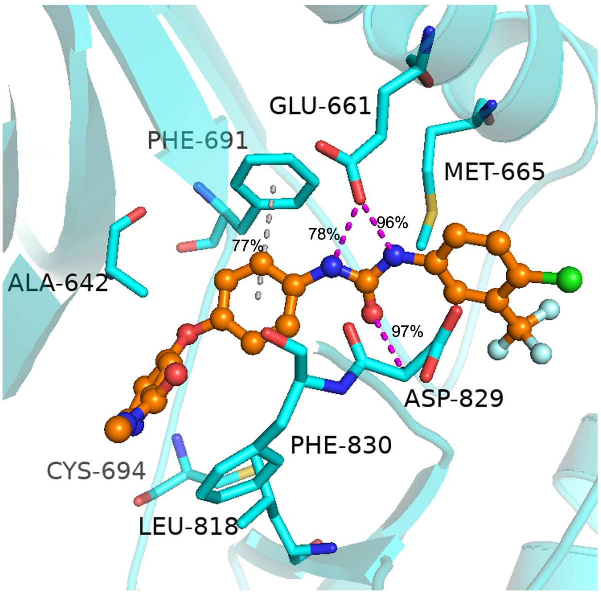

FIGURE S2: MD-predicted structure of the second scoring 4RT7-WT + sorafenib complex. 4RT7-WT backbone is shown as cyans cartoons. Sorafenib is shown as orange ball-sticks. Magenta dashed lines represent hydrogen bonds, and gray dashed lines represent Pi-Pi stacking interactions.

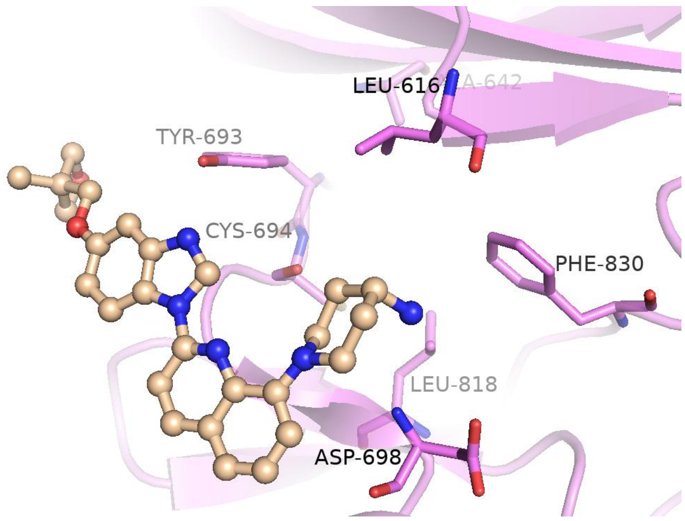

FIGURE S3: MD-predicted structure of the second scoring 6JQR-WT + crenolanib complex. 6JQR-WT backbone is shown as violet cartoons. Crenolanib is shown as wheat ball-sticks.

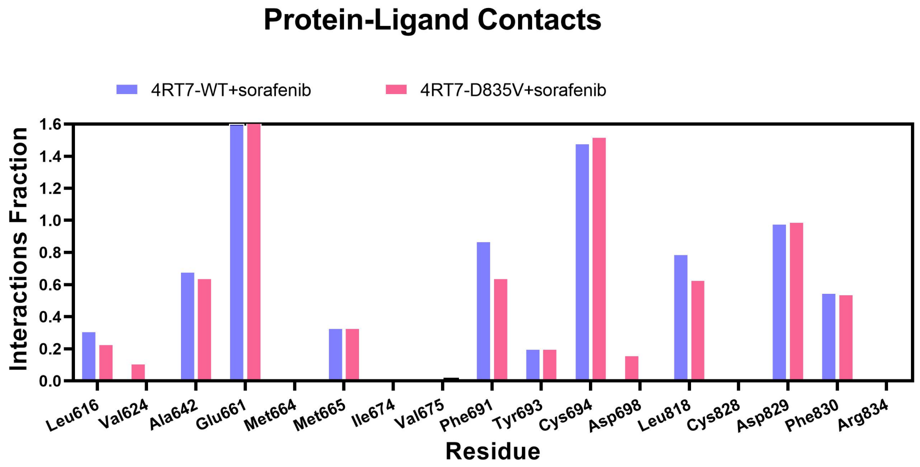

FIGURE S4: 4RT7 (WT and D835V) contact with sorafenib were monitored throughout the simulation, and the stacked bar charts were normalized over the course of the trajectory.

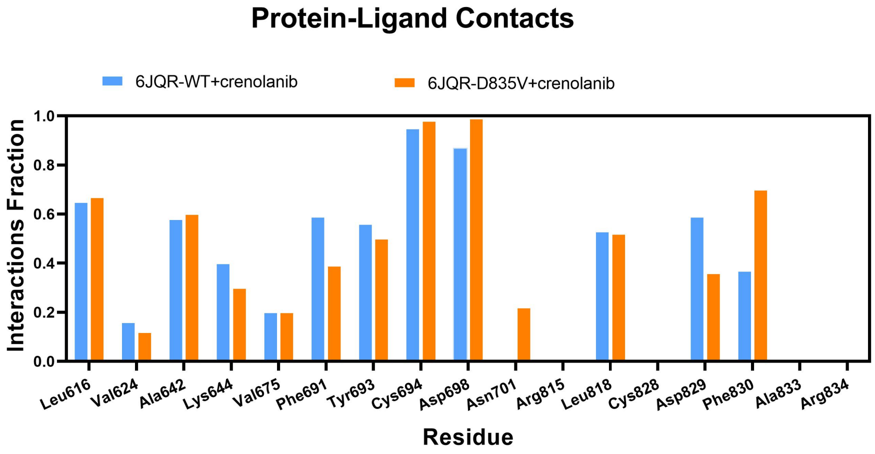

FIGURE S5: 6JQR (WT and D835V) contact with crenolanib were monitored throughout the simulation, and the stacked bar charts were normalized over the course of the trajectory.
